# Seven challenges in the multiscale modeling of multicellular tissues

**DOI:** 10.1002/wsbm.1527

**Published:** 2021-05-04

**Authors:** Alexander G. Fletcher, James M. Osborne

**Affiliations:** ^1^ School of Mathematics and Statistics University of Sheffield Sheffield UK; ^2^ Bateson Centre University of Sheffield Sheffield UK; ^3^ School of Mathematics and Statistics University of Melbourne Parkville Victoria Australia

**Keywords:** cell‐based modeling, developmental biology, multicellular systems biology, multiscale modeling, scientific computing

## Abstract

The growth and dynamics of multicellular tissues involve tightly regulated and coordinated morphogenetic cell behaviors, such as shape changes, movement, and division, which are governed by subcellular machinery and involve coupling through short‐ and long‐range signals. A key challenge in the fields of developmental biology, tissue engineering and regenerative medicine is to understand how relationships between scales produce emergent tissue‐scale behaviors. Recent advances in molecular biology, live‐imaging and ex vivo techniques have revolutionized our ability to study these processes experimentally. To fully leverage these techniques and obtain a more comprehensive understanding of the causal relationships underlying tissue dynamics, computational modeling approaches are increasingly spanning multiple spatial and temporal scales, and are coupling cell shape, growth, mechanics, and signaling. Yet such models remain challenging: modeling at each scale requires different areas of technical skills, while integration across scales necessitates the solution to novel mathematical and computational problems. This review aims to summarize recent progress in multiscale modeling of multicellular tissues and to highlight ongoing challenges associated with the construction, implementation, interrogation, and validation of such models.

This article is categorized under:

Reproductive System Diseases > Computational Models
Metabolic Diseases > Computational Models
Cancer > Computational Models

## Graphical abstract

This review summarizes recent progress in multiscale modeling of multicellular tissues and highlights ongoing challenges associated with the construction, implementation, interrogation, and validation of such models.

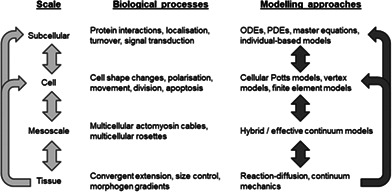

## 1 INTRODUCTION

How multicellular tissues self‐organize and remodel is a fundamental question in developmental biology with profound implications for tissue engineering and regenerative medicine (Sasai, 2013). Recently developed technologies such as single‐cell omics and light‐sheet microscopy (St Johnston, 2015) have resulted in a wealth of detailed, but isolated, descriptions of these processes. To truly comprehend such complexity, we need mathematical and computational models that can link observations and test hypotheses in a quantitative, predictive, and reproducible way (Sharpe, 2017). However, there are substantial methodological challenges associated with achieving these goals. This review aims to highlight these challenges and how they might be overcome.

**FIGURE 1:**
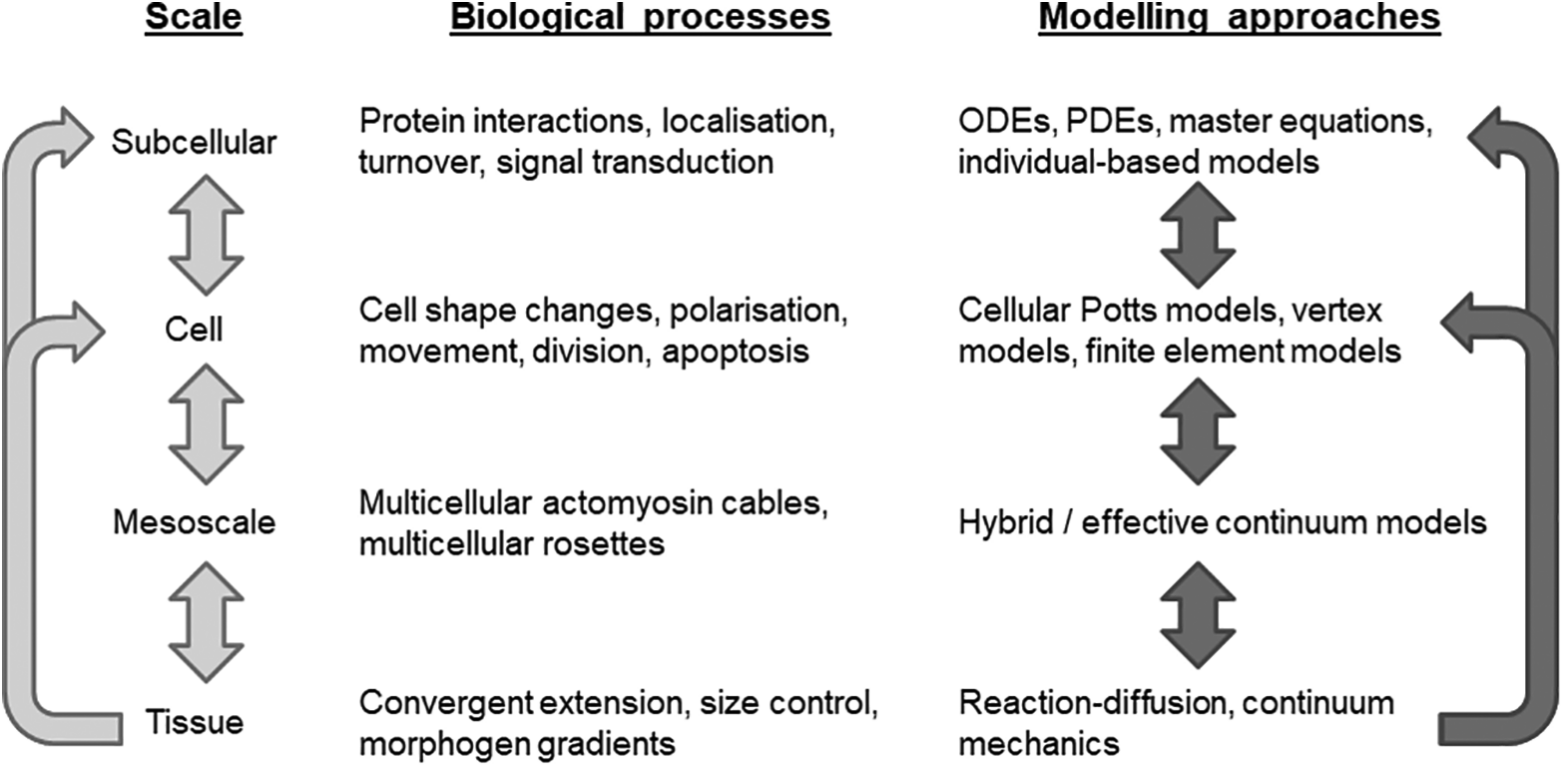
Modeling multiscale mechanisms of tissue self‐organization and remodeling. Hierarchy of spatial scales with associated biological examples and example modeling approaches. Light arrows show interdependence between scales. Dark arrows indicate where, in our view, new mathematical models are required that bridge scales

The growth and dynamics of multicellular tissues are multiscale in nature, involving tightly regulated and coordinated morphogenetic cell behaviors, such as shape changes, movement, and division, which are governed by subcellular machinery and involve coupling through short‐ and long‐range signals (Figure 1). A key challenge is to understand how relationships between scales produce emergent tissue‐scale self‐organization.

State‐of‐the‐art experimental approaches are revealing emergent behavior at different scales: super‐resolution microscopy allows quantification of complex, dynamic feedback interactions and subcellular localization underlying cell‐level patterning (e.g., see Liu & Keller, 2016); live‐imaging and force‐inference techniques uncover “mesoscale” structures, comprising groups of cells, driving tissue morphogenesis (Blanchard et al., 2019); while experimental techniques, such as ex vivo organoid cultures, offer a window into the contribution and timing of different signals to tissue self‐organization (Simunovic & Brivanlou, 2017). To fully leverage these techniques and obtain a more comprehensive understanding of the causal relationships underlying normal and abnormal tissue dynamics, computational modeling approaches are being developed that span multiple spatial and temporal scales, which couple cell shape, growth, mechanics, and signaling.

A common approach to modeling the dynamics of multicellular tissues is to treat the tissue as a continuous material and apply classical techniques from continuum mechanics and thermodynamics. While continuum models continue to generate valuable mechanistic insights into multicellular tissues, for example in characterizing the biomechanics of growing embryonic tissues (Ben Amar et al., 2019), they are less well suited to investigate individual cell level heterogeneities. On the other hand, the ever‐decreasing cost of computing power has facilitated the accelerated development of discrete agent‐based and multiscale modeling approaches, which more naturally allow investigation of the influence of intracellular and cellular level behaviors and heterogeneity in tissue‐scale phenomena. These approaches have recently facilitated successful interdisciplinary studies of emergent behaviors in developing tissues, such as identifying minimal sets of developmental rules for successful early embryonic development (Nissen et al., 2017); see also the recent special issue (Alber, Godin, et al., 2019).

Despite their utility, agent‐based and multiscale models of multicellular tissue remain technically challenging: modeling at each scale requires different areas of technical skills, while integration across scales necessitates the solution to novel mathematical and computational problems (Figure 1). In this review, we set out seven ongoing challenges in multicellular modeling, roughly in the order in which they may be experienced by someone developing a new model of this form: from model construction (1) and model calibration (2); to numerical solution (3) and software and hardware implementation (4); to model validation (5) and issues associated with model re‐use (data/code standards and benchmarks) (6) and comparing modeling assumptions and approaches (7). For each challenge, we summarize recent progress in specific applications as well as efforts to tackle the general case, highlighting one key open research question that we believe requires attention in future research.

When commenting on applications of agent‐based and multiscale modeling in the following, we focus primarily on animal developmental biology. This is a natural area in which to apply these modeling techniques, because of the availability of data across multiple spatial and temporal scales—from gene regulatory networks and signaling pathways to cellular interactions and larger‐scale morphogen transport processes—and the feedbacks between scales that underlies many emergent processes (Sharpe, 2017). We refer the interested reader to several recent reviews on the use of these modeling approaches in plant biology (Bucksch et al., 2017), cancer biology (Metzcar et al., 2019), synthetic biology (Gorochowski et al., 2020), and tissue engineering (Montes‐Olivas et al., 2019).

We do not claim that the challenges are exhaustive; nor do we claim that they are entirely unique to the field of multiscale modeling. Indeed, there remain general challenges associated with any interdisciplinary endeavor, such as how to communicate concepts and results among scientists with highly varied backgrounds. Nevertheless, our intention is to highlight progress and challenges in multiscale modeling in a way that is accessible to both practitioners (applied mathematicians, physicists, and computer scientists) and stakeholders (experimental biologists and clinicians), and to help inform the agenda for future computational multiscale modeling efforts.

## 2 CHALLENGE 1: MODEL CONSTRUCTION

In mathematical biology, modeling assumptions must be carefully tailored to the biological system of interest, the available data, and the scientific hypotheses. In the case of multiscale modeling, one must choose the level of complexity with which one describes subcellular processes, cellular interactions, and larger‐scale processes such as morphogen transport. This leads to our first ongoing challenge: *how to construct an appropriate model for a given problem*. Of course, this is a fundamental challenge in any mathematical modeling endeavor, and a balance must be struck between precision, generality, and realism.

A variety of multiscale modeling approaches have been developed for multicellular tissues (Fletcher et al., 2017). These approaches generally either discretise space into a regular (Lehotzky & Zupanc, 2019) or irregular (Scianna & Preziosi, 2016) lattice, or allow cells to move continuously in space (Drasdo & Höhme, 2005). Such approaches also vary in their geometric description: cells may be treated as point‐like particles or prescribed shapes such as ellipsoids (Xu et al., 2008); or cell shape may be more complex and dynamic, evolving according to a balance of forces (Farhadifar et al., 2007) or stochastic fluctuations (Graner & Glazier, 1992). There can also be increased geometric complexity moving from two‐dimensional to three‐dimensional representations, such as a larger number of possible cell interactions and configurations (Okuda et al., 2015). Another distinction can be made based on the level of biophysical complexity used: considering cell surface mechanics only (Magno et al., 2015); or including subcellular heterogeneity such as the presence and mechanical role of the nucleus and other organelles. The latter include the subcellular element model introduced by Sandersius and Newman (2008), which has recently been applied to epithelial tissue dynamics, for example (Nematbakhsh et al., 2017, 2020).

Several modeling challenges remain. For example, there is increasing evidence for the importance of subcellular spatial patterns of protein complexes for the generation of cellular, and hence tissue, asymmetry (Strutt et al., 2011). This necessitates the development of more detailed models that include processes such as directed vesicular transport, trafficking and recycling, and cell shape changes. Other challenges include how to model cellular projections such as cytonemes and their role in cell–cell communication (Bressloff & Kim, 2019; Rosenbauer et al., 2020) and the formation and functional roles of supracellular structures such as multicellular rosettes (Trichas et al., 2012) and supracellular actomyosin cables (Tetley et al., 2016). Overall, a major challenge lies in the link between biochemistry in these models (Mak et al., 2015; Velagala et al., 2020). Descriptions of mechanics have traditionally been very simple in this area; as models adopt more sophisticated mathematical treatments growth, morphoelasticity, and mechanotransduction, we face the prospect of more complex models that may be more complex simulate and calibrate, but are increasingly physically grounded and, in principle, more amenable to experimental validation).

## 3 CHALLENGE 2: MODEL CALIBRATION

Having arrived at a set of modeling assumptions, one is faced with the issue of how to choose appropriate parameter values and initial conditions. This leads to our second ongoing challenge: *how to calibrate a multiscale model against data*. By “calibration,” we refer both to inference of model parameters and their associated uncertainty, and calibration of initial or boundary conditions (e.g., initial cell and tissue geometry), as discussed below. In an ideal world, we would have enough data at each level of a model to fully calibrate it. In practice, we may require various techniques to accommodate data at each level that are quantitative, qualitative, or unavailable (Pargett & Umulis, 2013).

Initial conditions are specified based on our biological understanding. In some cases, this can be straightforward, for example when modeling tissue development from a single stem cell (Atwell et al., 2015). This ad hoc approach works well if there are initially few cells, or if the initial state is highly conserved. Another common approach is to evolve the system to a dynamic equilibrium that is not sensitive to the choice of initial condition, as in the case of many homeostatic tissues such as the skin (Li et al., 2013) or gut epithelium (van Leeuwen et al., 2009). For developing tissues undergoing morphogenesis, an alternative approach is to derive initial conditions such as cell packing directly based on microscopy images (Iber et al., 2016) or via extracted summary statistics (Vicente‐Munuera et al., 2019). Here, one should bear in mind variability in structures and it may be more important to capture essential patterns and conserved properties of tissue organization rather than use a specific example.

As well as establishing initial conditions such as cell and tissue geometry, one must arrive at values for model parameters. Typically, parameters cannot be measured directly but must be inferred from experimental data. Where datasets are available for parameter estimation, approximate Bayesian computation (ABC) and its variants are increasingly used, since the likelihood of a multiscale model is typically neither analytically nor computationally tractable (Kursawe et al., 2018; Lambert et al., 2018). These approaches compare model outputs with experimental data and accept parameter values for which these are sufficiently close. The inference process can be streamlined by the use of recent software tools such as pyABC (Klinger et al., 2018) and Pakman (Pak et al., 2020). These techniques can also be used to perform model selection when exploring two or more competing biological hypotheses in silico; we discuss the comparison of multiscale models in more detail when discussing Challenge 7. More general local and global sensitivity analysis methods have also been developed to assess to which parameters a model is most sensitive, and to identify the effects of uncertainties in parameter values on its output, and these have been applied to multicellular models (for an example approach, see [Boas et al., 2015]). A common issue faced in multiscale modeling is whether parameters are uniquely identifiable from experimental data in the first place, and the development of efficient techniques for testing identifiability remains an active research area (see e.g., Kreutz, 2018).

Several challenges remain when specifying initial and boundary conditions and estimating parameters of multiscale models. In particular, more tailored and efficient parameter inference techniques are needed that rely less heavily on repeated simulations of computationally expensive models, for example by exploring parameter space more intelligently and by exploiting “low‐fidelity” or surrogate modeling (a recent, relevant domain‐specific example of this is Prescott & Baker, 2020 ). Where simulation outputs are intended to inform decision‐making, greater use of uncertainty quantification techniques is needed, for which software tools are increasingly available; in this regard, for further discussion of VVUQ and related concepts, see Richardson et al., 2020. More generally, the integration of machine learning approaches with multiscale modeling holds significant promise (Alber, Buganza Tepole, et al., 2019; Peng et al., 2020).

## 4 CHALLENGE 3: NUMERICAL SOLUTION

Due to their complexity, multiscale models of multicellular tissues are typically analytically intractable and must be solved numerically. This leads to our third challenge: *how to solve multiscale models efficiently, stably and accurately*. Their numerical solution remains challenging since the coupling of processes or sub‐models across scales can make it harder to avoid numerical instabilities, and many such models include parameters of numerical approximation; for example, thresholds for changes in cell topology (Fletcher et al., 2013). Thus, we need to be aware of any impacts that numerical implementation choices may have on model predictions (Kursawe et al., 2017).

Often, subcellular processes such as protein production, degradation, or complex formation may be assumed to evolve on a different timescale to cellular level processes such as division and movement, or tissue‐scale processes such as long‐range diffusion. Therefore, based on quasi‐steady state modeling approximations or similar, multiscale models may be decomposed into subsystems that can then be solved numerically over discrete time steps during which slower evolving subsystems are held constant.

For deterministic systems, traditional approaches make use of forward Euler integration schemes (Osborne et al., 2017); however, higher‐order explicit (or implicit) numerical solvers are more computationally intensive, and require modifications to account for cell death and division changing the size of the dynamical system over each time step, but allow larger time steps to be used while maintaining stability (Atwell, 2016). For some cell‐based models, numerical methods must be carefully chosen to respect physical conservation laws (Cooper et al., 2017). Efficient numerical techniques have also been developed to simulate stochastic reaction–diffusion processes in spatially complex or evolving domains (e.g., Drawert et al., 2012). At the cellular level, algorithms have been developed to simulate the evolution of stochastic models such as the cellular Potts model efficiently while forbidding physically unrealistic phenomena such as cell fragmentation (Durand & Guesnet, 2016). Several efforts have also been made to develop efficient solvers that couple cell‐based models and tissue‐scale processes such as nutrient transport (Ghaffarizadeh et al., 2016), morphogen signaling (Smith et al., 2012), and fluid flow (Grogan et al., 2017; Osborne & Bernabeu, 2018), based on finite element, finite volume, finite difference, and lattice Boltzmann methods.

Looking ahead, major challenges in numerical solution include the application of adaptive methods, which allow spatial or temporal discretizations to vary according, for example, to the amount of cell movement or proliferation in a domain. Numerical methods should be chosen to ensure convergence and accuracy, a posteriori error analysis techniques (Cangiani et al., 2018) or multiscale analysis (Whiteley, 2006) offer an exciting avenue for optimizing numerical accuracy and stability. This also leads to the prospect of models that allow one to change the level of spatial complexity when and where needed, for example by using a cell‐based description in regions where the bulk of growth occurs, and a continuum description elsewhere (Kim et al., 2007). The assessment of different numerical techniques brings us to the issue of benchmark problems, which forms the focus of Challenge 6.

## 5 CHALLENGE 4: SOFTWARE AND HARDWARE IMPLEMENTATION

**FIGURE 2:**
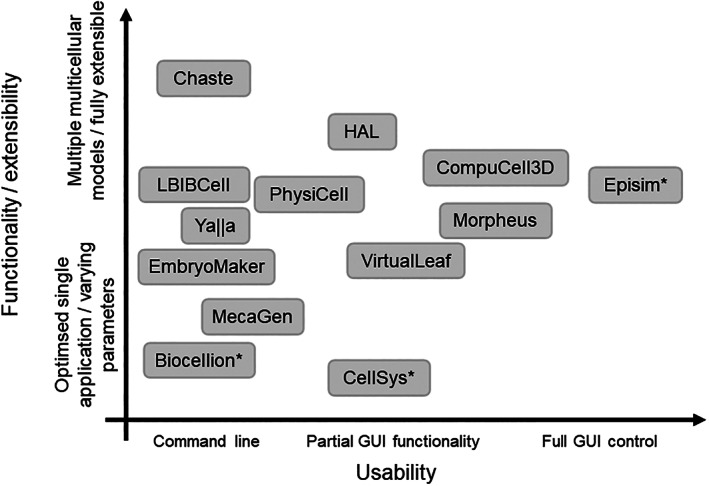
Summary of the major existing software tools for multiscale modeling of multicellular tissues based roughly on their usability and functionality/extensibility. We define usability by the level of graphical user interface (GUI) support for model construction, visualization and analysis; we do not account for ease of use in any greater detail, since this depends strongly on user background and experience. We define functionality/extensibility in terms of the range of possible multicellular modeling assumptions that may be implemented; we do not account for other factors such as use of markup languages to import submodels, nor level of HPC or GPGPU support, since this can change rapidly. Asterisks denote tools that are not open source. References for these tools are as follows: Biocellion (Kang et al., 2014); CellSys (Hoehme & Drasdo, 2010); Chaste (F. Cooper et al., 2020); CompuCell3D (Swat et al., 2012); EmbryoMaker (Marin‐Riera et al., 2015); Episim (Sutterlin et al., 2013); HAL (Bravo et al., 2020); LBIBCell (Tanaka et al., 2015); MecaGen (Delile et al., 2017); Morpheus (Starruß et al., 2014), PhysiCell (Ghaffarizadeh et al., 2018); VirtualLeaf (Wolff et al., 2019); ya||a (Germann et al., 2019)

If every researcher implemented their own model from scratch, we would have unnecessarily many implementations at a massive overall time cost. The solution to this is to share and re‐use computational implementations, which leads to our fourth ongoing challenge: *how to fully exploit modern software and hardware architectures*. Multiple numerical libraries and software tools have been developed for building, running, and interrogating multicellular models. These range from user‐friendly GUIs with limited functionality intended for use by biologists to more extensible software libraries, which require significant programming skills to utilize (Figure 2).

A key recent development has been the increased use of parallelization and high‐performance computing (HPC). Two common use‐cases for HPC, typically requiring different parallelization approaches, are: to run a large number of simulations, each at reasonable computational cost (e.g., when performing parameter sweeps); and to run a small number of simulations, each at significant computational cost (e.g., when simulating the collective dynamics of tens or hundreds of thousands of cells).

The first use‐case is “trivially” parallelizable, in that multiple instances may be run independently on multiple processors in parallel. In contrast, the second use‐case requires one to partition a simulated model into smaller components, for example through spatial decomposition, that are simulated in each processor. Parallel interfaces such as MPI (for distributed memory devices) and OpenMP (for shared memory devices) are then used to communicate information between processors. Such algorithms have been developed and publicly released, for example, for the cellular Potts model (Chen et al., 2007) and for off‐lattice cell‐center models (Harvey et al., 2015). Further speedups may be obtained through the use of GPGUs, for example through using CUDA or OpenCL; again, published examples include released implementations of the cellular Potts model (Tapia & D'Souza, 2011) and off‐lattice cell‐center models (Germann et al., 2019).

Several software and hardware challenges remain. When developing, designing, and refining software tools for multiscale modeling, one must carefully balance functionality and usability; such tools are unlikely to be taken up by non‐specialists unless barriers are further lowered, for example through the greater use of Graphical User Interfaces (GUIs) and Application Programming Interface (APIs). Modern computing resources, such as cloud computing, should also be more fully exploited; a laudable example of work toward this is xml2jupyter, which provides a web‐based interface for simulations (Heiland et al., 2019). A big issue is that each of these efforts takes a large commitment in both time and effort, and the challenge is to find resources to work on GUIs and APIs. Finally, there remains remarkably little in the way of cross‐validation of different model implementations; we return to this point when discussing Challenges 6 and 7.

## 6 CHALLENGE 5: MODEL VALIDATION

A multiscale model may be used to leverage existing data and obtain a more comprehensive understanding of the causal relationships underlying tissue self‐organization and remodeling. If a model reproduces key observations in a robust and reproducible way, then a natural next step is to use the model to generate experimentally testable predictions for what we should observe under different circumstances, for example, when the system undergoes a genetic or mechanical perturbation. By testing these predictions, we can seek to demonstrate the model's predictive functionality and validate its model assumptions through qualitative and quantitative comparisons of model outputs to data. This leads to our fifth ongoing challenge: *how to validate multiscale models against data?* This strongly relates to the issue of model selection and parameter collaboration discussed in Challenge 1 and 2, but is typically encountered later in the modeling process. In each case, we typically have access only to partial information about the system at each spatial or temporal scale, and so we may need to resort to validating separate model components individually (Viceconti et al., 2020).

Testing model predictions, like estimating model parameters (see Challenge 2), often involves using various different types and sources of experimental data. Subcellular processes, such as protein dynamics, may be compared—at least semi‐quantitatively—against experiments on isolated cells or on in vitro cell cultures (Tan et al., 2012), with due care for possible discrepancies with the in vivo setting. At the cellular and tissue level, rheology experiments, laser ablations, and non‐invasive force inference techniques can all be used to interrogate how tissues respond to applied stresses (Sugimura et al., 2016) and compare these measurements with model predictions. Cell tracking can also be used to match cell velocities and other summary statistics. In this context, recent work demonstrating the pitfalls of inferring cell‐level heterogeneity from such data (Schumacher et al., 2017) has implications for inferring parameters from the “wrong” model; we return to the issue of comparing models and modeling assumptions when discussing Challenge 7.

Another challenge is to optimize our use of information at different scales. To what extent can we validate a given multiscale model, and which experiments will yield the greatest information for this purpose? This requires advances in our statistical understanding of how best to use information at different scales. If data at certain scales are limited, then one possible strategy is to employ mathematical (e.g., asymptotic or equation‐free) methods to obtain a coarse‐grained model (whose parameters are related to the full model) that may be more amenable to efficient simulation, parameter sweeping, and qualitative mathematical analysis. Continuum coarse‐grained models have been derived from to lattice‐based models (Johnston et al., 2012) and cellular Potts models (Alber et al., 2007), as well as off‐lattice cell‐center models (Murray et al., 2012) and vertex models (Fozard et al., 2010). It remains an open question to what extent validation of such coarse‐grained models might help inform us about a full model.

## 7 CHALLENGE 6: DATA/CODE STANDARDS AND BENCHMARKS

There are myriad simulation tools, as mentioned above. This is unlike fields like molecular dynamics where one or two libraries (Hess et al., 2008) are used by the majority of researchers or “subcellular” systems biology where the predominant modeling formalism is shared across the field (Hucka et al., 2003). We need to assess how simulation results compare between different software and hardware implementation. This leads to our sixth ongoing challenge: *how to develop standards and benchmarks for multicellular implementation comparison*.

There are ongoing discussions in the wider field of computational biology regarding the need for model replicability and reproducibility (Grüning et al., 2018; Lewis et al., 2016). In the case of multiscale modeling of multicellular tissues, model construction, exchange and re‐use remain hampered by the lack of easily shareable model definitions, for example in the form of a fully declarative model definition language. SBML and CellML have largely solved this problem in the context of reaction kinetics, and have associated APIs and model repositories, but have limited functionality for spatial models; some initiatives are ongoing in this area, such as the MultiCellML project. Many multicellular tools have support for SBML specification of subcellular processes, for example, CompuCell3D (Swat et al., 2012), Chaste (Romijn et al., 2020), and Morpheus (Starruß et al., 2014). Another aspect of this is separating out the definition of the biological process from the definition of any in silico experiments or perturbations, allowing easier comparison of model behavior with experimental data. Relatively little work has been done on this (Cooper & Osborne, 2013) compared to more mature areas of systems biology such as cardiac electrophysiology (Cooper et al., 2016).

Several challenges remain in this area. In particular, we must work out how to improve standards, reproducibility, and interoperability among new and existing software tools (Macklin, 2019). The first step of this process is to develop a set of profiling benchmark problems for each model type. This will both help inform the standards required for model specification and provide an objective means for tool comparison. There may be lessons to be learned from other domains such as astrophysics, weather/climate modeling, and molecular dynamics, as well as other areas of systems biology such as cardiac electrophysiology (Niederer et al., 2011).

## 8 CHALLENGE 7: COMPARING MODELING ASSUMPTIONS AND APPROACHES

As outlined above, there are many ways to model the dynamics of individual cells and their interactions within tissues, to solve a model numerically, and to implement this in the computer. This leads to our final ongoing challenge: *how to assess robustness of model behavior to choice of modeling paradigm*. This is of particular relevance to biomedical applications, if predictions are to inform clinical practice.

It can be difficult to disentangle differences in model behavior arising from algorithmic, software, and hardware implementation. However, some progress has been made on aspects of this issue. Simulation studies have been used to compare different model components (mechanical assumptions and constitutive equations) for particular classes of cell‐based models, such as cell‐center models (Mathias et al., 2020; Pathmanathan et al., 2009) and vertex models (Fletcher et al., 2013). Efforts have also been made to compare and contrast five competing cell‐based model paradigms, including lattice‐based and off‐lattice models, in a consistent computational framework (Osborne et al., 2017), to help elucidate where one may expect to see qualitative differences between model behaviors. Four exemplar simulations were chosen to compare the models, which represent facets of development: adhesion; proliferation, death, and differentiation; short‐range signaling; and long‐range signaling. At a tissue level, each of the modeling paradigms investigated was able to implement these processes and, outside a few edge cases where artifacts were introduced, the tissue level behavior was qualitatively the same. A natural extension of this work is to compare models across different software implementations, such as those discussed in Challenge 6. This is feasible in principle, although one needs to ensure the same model definition and equivalent method of numerical solution (i.e., different integrators to within some tolerance), which is difficult in the absence of model standards discussed above.

In addition, the challenge of defining a suitable set of pedagogical benchmark biological problems with which to simulate one or more models remains. Examples for comparison need to be chosen to identify and highlight similarities and differences between model paradigms. Despite recent workshops and collaborative modeling endeavors we are still only starting the journey of model comparison and future progress on this challenge will require support from the whole multicellular modeling community.

## 9 CONCLUSION

In this paper, we have presented seven open challenges associated with multiscale modeling of multicellular tissue dynamics. These challenges range from the mathematical (how to model processes across multiple scales, see Challenge 1), the numerical (how to make use adaptive numerical methods and HPC, see Challenge 4), and the statistical (how to infer multiscale model parameters from partial information on processes at each scale, see Challenges 2 and 5) to the computational (how to unambiguously define such models in a shareable format, see Challenges 6 and 7) and even societal (how to collectively arrive at standards and benchmarks, see Challenge 6). In our view, overcoming these challenges will require a significant community‐driven effort in order to move such modeling approaches to a point where they can be a standard tool for stem cell and developmental biologists and tissue engineers alongside experimental approaches. Addressing these challenges, particularly those associated with quantifying uncertainty in model predictions, is even more important if such approaches are to become a greater part of clinical research and decision‐making, as is starting to be seen for example in mathematical oncology (Rockne et al., 2019).

## AUTHOR CONTRIBUTIONS

**Alexander Fletcher:** Conceptualization; investigation; methodology; visualization; writing‐original draft; writing‐review & editing. **James Osborne:** Conceptualization; investigation; methodology; visualization; writing‐original draft; writing‐review & editing.

## RELATED WIREs ARTICLES

Multiscale models of thrombogenesis

Multiscale modeling methods in biomechanics

Multiscale modeling of vertebrate limb development

Multiscale modeling for biologists

Agent‐based models in translational systems biology

## Data Availability

Data sharing is not applicable to this article as no new data were created or analyzed in this study.
